# Dynamic Analysis of DNA Damage by Flow Cytometry and FISH

**DOI:** 10.1100/tsw.2006.112

**Published:** 2006-05-15

**Authors:** Demetrios P. Matthopoulos

**Affiliations:** Department of Environmental and Natural Resources Management, Ioannina University, Seferi 2, Agrinio 30100, Greece

**Keywords:** micronuclei, human chromosomes, flow cytometry, FISH, noxious agents

## Abstract

The micronucleus assay, developed to assess DNA damage induced by noxious agents, supplies information on whether the damage is due to clastogenic or aneugenic action. Although it is the test that can be used to assess agents' toxicity, it cannot provide information on the molecular events that result in the induction of micronuclei. To study the molecular events, the combination of both microscopic and analytical techniques is required. Flow-sorting induced micronuclei, based on their DNA content, in combination with chromosomal FISH and other molecular techniques, may provide information on these events.

### INTRODUCTION

Noxious agents, natural ones, surround living systems on earth from the very beginning of life. Life evolved and developed under their effect. The evolutionary pressure developed by these agents, natural conditions, supplied living organisms with the appropriate defensive mechanisms. Can we consider earth's environment as a hostile one or not? To do so, we have to understand the course of evolution from the very beginning until today and, furthermore, into life's future evolution.

The onset of the species *Homo sapiens* stamped a new era in species evolution. In particular, by the time of technological development, man started to intervene with nature in such a way that irreversible changes have been introduced into natural conditions.

This human intervention with nature, being the result of technological development, introduced a new series of noxious agents. From their initial introduction to nature, these new agents exert their effect on living organisms. The rate by which they are introduced into the environment is such that nature may require longer periods than before to readapt itself and further develop its defensive mechanisms.

Man introduced chemicals that, along with radiation, are responsible for numerous negative side effects such as biochemical malfunction or genetic instability[1,2]. The genetic instability can either be the result of aneugenic or clastogenic effects[3,4]. In aneugenic action, noxious agents interfere with the mitotic apparatus, resulting in the disorientation of segregating chromosomes that result in the cytoplasm instead of being incorporated into the daughter nuclei. In clastogenic action, noxious agents interfere with DNA, causing breaks that result in acentric chromosomal fragments that are disoriented during mitosis.

Analytical biochemical, histochemical, and cytogenetic techniques have been developed for studying the possible side effects of such noxious agents[5,6,7,8,9,10]. These techniques provide information that helps us to understand their way of action and to plan possible treatments against their effects. Detailed information can be revealed by combining observations from several techniques, leading us to assess the risk of human exposure to these agents[3].

Their intervention on the genetic consistency of organisms is variably expressed in different tissues. Highly regenerating tissues, such as hematopoietic tissue and dermis, are subjected to changes that usually ease off due to the high turnover of their cells. Changes to other tissues may result in either cell death or neoplastic development. In multicellular organisms, individual cell death has no negative result, while neoplastic development is usually fatal. The programmed cell death, apoptosis[11], has been shown to be directly modulated by several oncogenes and tumor-suppressor genes[12]. Oncogenes such as bcl-2 and viral products such as adenovirus E1B or SV40 large T antigen induce neoplasia via apoptotic inhibition[13,14]. DNA damage resulting in chromosomal rearrangements is one of the events that lead to cancer development[15,16]. In apoptosis, DNA programmed fragmentation is the main event. In both cases, cancer development and apoptosis, disoriented nuclear material during mitosis is dispersed in the cytoplasm in the form of micronuclei, giving rise to aneuploidy[17]. DNA repair mechanisms in these two processes, as well as in genotoxic conditions, fail to respond.

It becomes obvious that the clastogenic or aneugenic potential of noxious agents, such as chemicals and radiation[18,19,20,21,22,23], is of considerable importance. To evaluate their potential for inducing chromosome breaks (clastogenic action) or poisoning mitotic spindle apparatus (aneugenic action), the micronucleus (MN) assay was introduced for both *in vivo* and *in vitro* studies[24,25,26]. During the last few years, molecular techniques were introduced in combination to the micronucleus assay for the molecular analysis of genotoxicity[27,28,29,30,31].

Fluorescence *in situ* hybridization (FISH), carried out according to Pinkel et al.[32], introduced by Miller et al.[33] for the classification of induced micronuclei in murine erythrocytes, and advanced by Schriever-Schwemmer and Adler[34] proved to be a powerful technique. It can be applied to all cell types containing micronuclei[35,36]. Centromeric and telomeric probes were used in FISH studies in an attempt to elucidate the aneugenic or clastogenic way of action of noxious agents on DNA[27,28,29,30,31,37]. Although such probes give us the ability to distinguish between aneugenic and clastogenic action, they do not supply information on the molecular events preceding these actions. The analysis of these events requires the design of specific probes. Such probes can easily be designed if the DNA contained in micronuclei was known. Thus, the isolation of micronuclei could be informative in this direction. There are two methods for such isolation. The one is LASER capture microdissection (LCM) and the other is flow cytometry (FC).

LCM[38,39,40] is a powerful microscopic technique that provides material for molecular analysis. However, it is time consuming because one needs enough material to work with. On the other hand, FC[41,42] (being a powerful method for the analysis of cell types and subcellular constituents) proved to be the technique of choice for the analysis and isolation of chromosomes based on their DNA content and not on their size. Isolated chromosomes subsequently can be used for molecular cytogenetic analysis. Rearranged chromosomes, because their DNA content is different from their normal counterparts, are easily identified and isolated for molecular analysis[43]. FC, early enough, was used for the enumeration and analysis of micronuclei induced by various agents[18,20,22,31]. By itself, it cannot provide information on the molecular events that lead to the induction of micronuclei, but if combined with analytical techniques, it can be very informative. Flow analyzing and isolating micronuclei may be the method of choice for the study of the molecular events that result in their induction by noxious agents.

The aim of the present work is to evaluate the possibility of using flow sorting for the subsequent molecular analysis of induced micronuclei. For this purpose, human neoplastic cells were used in order to analyze their rearranged chromosomes for subsequently sorting their chromosomal markers.

## MATERIALS AND METHODS

### Cell Lines

The human neoplastic cell lines SW620, HEMI 2, and HEMI 5, supplied by Dr. M. Meuth of Cancer Research U.K., were used in the present study. They have derived from adenocarcinomas developed from secondary metastasis of colorectal carcinomas.

They are highly tumorogenic cells grown in monolayer cultures in the presence of Dubelcco's modified Minimal Essential Medium supplemented with 10% fetal bovine serum. Subconfluent cultures were maintained at 37°C in a 5% $CO_2$ humidified atmosphere.

The lymphoblastoid cell line GM1416B with a 48XXXX karyotype was used as control throughout the experiments.

### Chromosome Preparation

Chromosomes were prepared as previously described[43]. In brief, actively growing cultures were treated with colcemid at concentrations of 0.1–0.2 µg/ml of culture medium for a period of 12–16 h. Cells were collected from mitotic shakeoffs by centrifugation at 800 rpm at room temperature. Pellets containing approximately $10^7$ cells were collected in 50-ml centrifuge tubes and hypotonic treated with 2.5 ml of 40 m*M* KCl, 0.2 m*M* spermine, and 0.5 m*M* spermidine for 20 min at room temperature. Thus, treated cells were collected by centrifugation at 800 rpm for 10 min and resuspended in 1 ml of ice cold, chromosome isolation buffer. The suspended cell pellet was left undisturbed in ice for up to 1 h and slowly vortexed for 10 sec to disrupt cellular integrity and disperse nuclei and chromosomes. Chromosome suspensions were kept at 4°C until they were used for flow cytometric analysis and sorting. Before cytometric analysis, chromosomes were stained with 2.8 µg/ml of Hoechst 33258 and 75 µg/ml of Chromomycin $A_3$ for 2 h at 4°C.

### Flow Cytometry

Flow cytometric analysis and, subsequently, isolation was performed using a Becton-Dickinson FACStar-Plus analyzer-sorter equipped with two 5W+UV Argon ion LASERs. The primary laser was tuned at 262 nm to excite Hoechst 33258 while the secondary laser was tuned at 457 nm to excite Chromomycin $A_3$. Photomultipliers' voltage and gain were continuously monitored and readjusted in order that the chromosome profile be equally distributed on the computer monitor. Under these conditions, intact nuclei are located off to the upper right corner of the monitor, while normal and marker chromosomes are identified in the lower left corner. Increasing voltage and gain, marker chromosomes were better analyzed and, after applying sorting windows around particular chromosomal areas, could be isolated.

## RESULTS

All three neoplastic cell lines, SW620, HEMI 2 and HEMI 5, have highly abnormal karyotypes with many marker chromosomes. Some of these marker chromosomes are larger than chromosome #1 and others smaller than #22. Those larger than chromosome #1 and several other marker chromosomes are rearranged ones. Those smaller than chromosome #22 and several minute ones could easily be either rearranged or chromosomal breakoffs.

Fig. 1 (a, b, c) depicts the chromosomal flow analysis of the SW620, HEMI 2, and HEMI 5 cell lines, respectively, compared to the near-normal flow karyotype of the human 4X cell line GM1416B (Fig. 1d). The GM1416B flow karyotype has been annotated, indicating the positioning of normal chromosomes. It is obvious that the flow karyotypes of the three neoplastic cell lines deviate from the normal one.

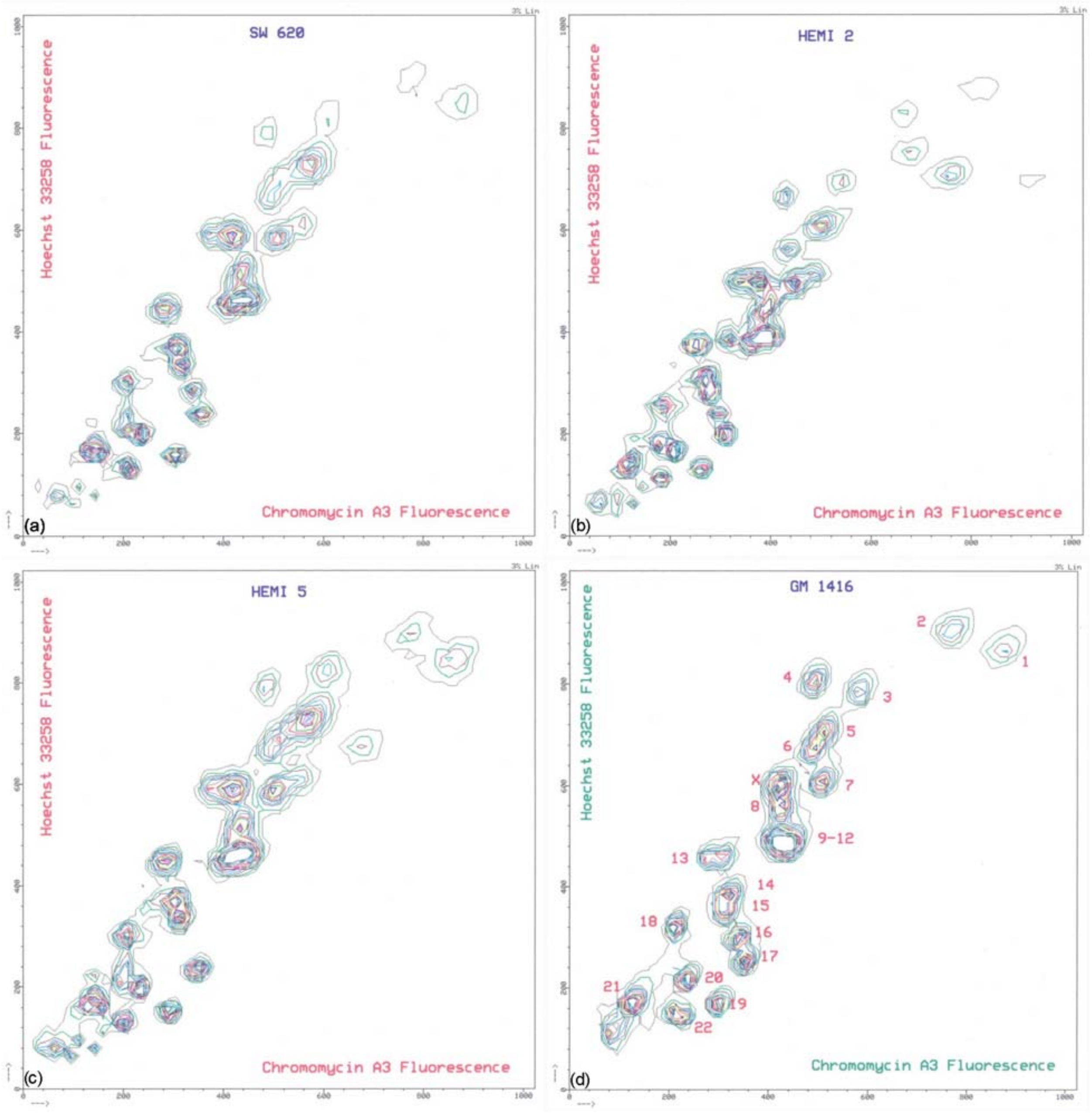

**FIGURE 1.** Flow karyotypes of the various cell types: (a) – (c) SW620, HEMI-2, and HEMI-5 neoplastic cell lines, respectively, while (d) GM1416B, 4X lymphoblastoid cell line. Annotations in (d) indicate the normal positions of the various chromosome pairs. Chromomycin $A_3$ vs. Hoechst 33258 fluorescence.

By increasing or decreasing the voltage and gain of the photomultipliers, we can either distinguish those larger chromosomes than the normal #1 or those smaller than the normal #22 marker chromosomes of the three neoplastic cell lines, respectively.

Fig. 2 (a, b, c) depicts the neoplastic flow karyotypes at photomultiplier voltage and gain high enough to segregate those smaller than the normal chromosome #22 marker chromosomes. Several small marker chromosomes in all three neoplastic cell lines can easily be identified.

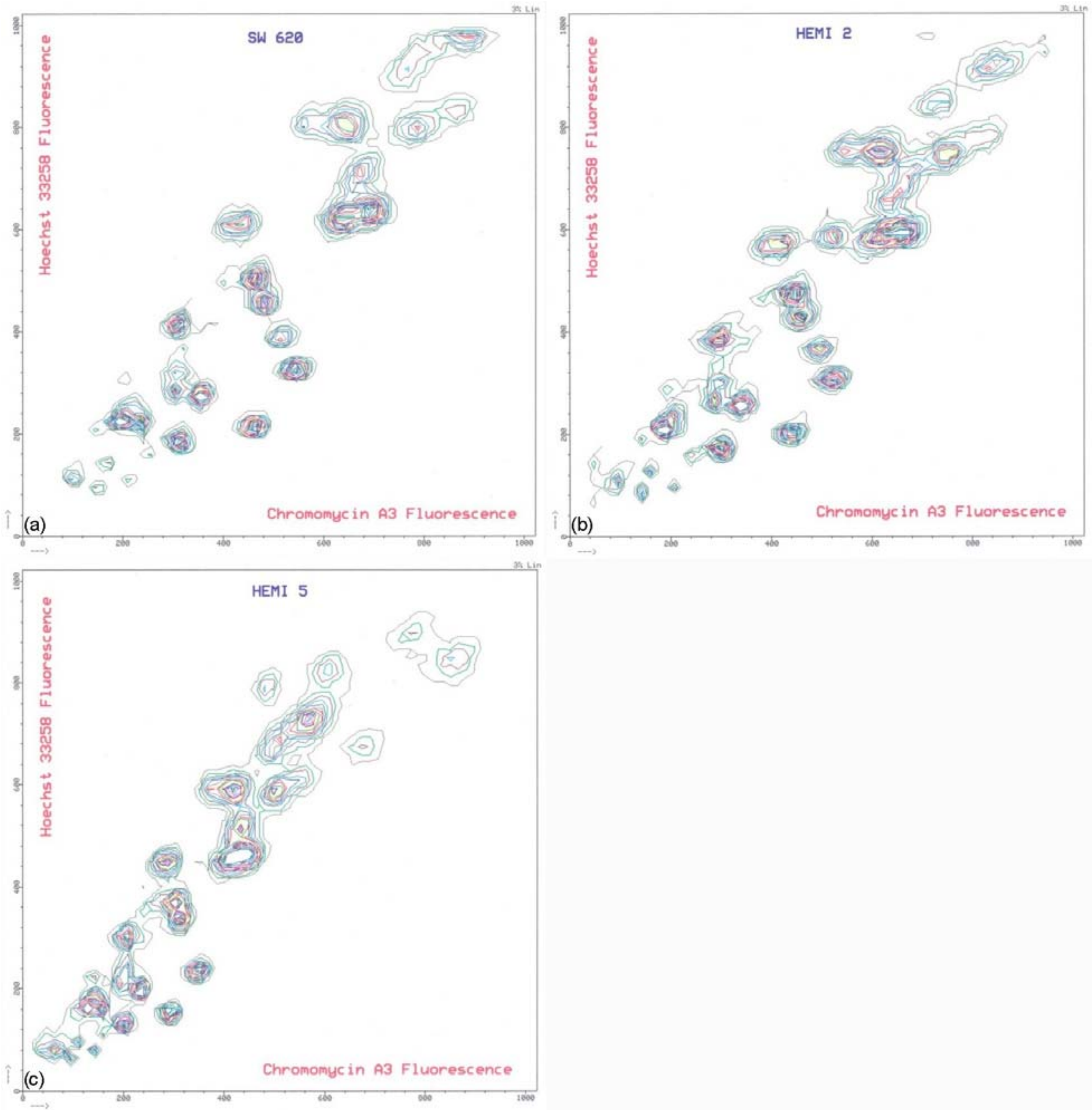

**FIGURE 2.** Flow karyotypes of the neoplastic cell lines at increased photomultiplier voltage and gain in order for the small marker chromosomes to be revealed. (a) – (c) SW620, HEMI-2, and HEMI-5 neoplastic cells, respectively. Chromomycin $A_3$ vs. Hoechst 33258 fluorescence.

By organizing sorting windows around defined phasmatogram areas, we can sort particular marker chromosomes no matter their size, as chromosome analysis is based on the amount of DNA contained in each chromosome.

### DISCUSSION

Exposing organisms to noxious agents may result in DNA damage. The extent of damage depends on parameters such as agent concentration, duration of exposure, age of organism, etc. The damage may range from minor reparable DNA alterations up to extensive ones that may result in genetic instability and organism death. Diagnostic methodologies have been developed to assess the extent of damage at early stages in order to react appropriately.

Initially, microscopic techniques were applied that provided morphological and statistical data. Various indexes, based on such techniques, were introduced for the distinction between aneugenic and clastogenic effects of environmental parameters on DNA integrity[44,45]. These indexes provide general information. Assessment based on such data is valuable; however, information on the molecular events that take place cannot be revealed. Such information requires the introduction of dynamic cytogenetic and molecular techniques. In the last few years, techniques such as FC, FISH, and single nucleotide polymorphism (SNP) were introduced in the study of the genetic instability induced by noxious agents[18,20,22,27,28,29,30,31]. The introduction of FC in the above studies made the analysis easier and the statistical evaluation more reliable, while the introduction of FISH using centromeric DNA probes[33,34] provided some information on the chromosomal constituency of the induced micronuclei. However, although they supply valuable information, these techniques are not as informative as they should be, unless they are combined with more analytical molecular techniques.

Chromosomal FISH, derived from isolated rearranged chromosomes[43] and accompanied with molecular techniques, may help us to elucidate the molecular events that are responsible for the induction of cytogenetic abnormalities or neoplastic development.

Cell sorters can easily analyze and separate rearranged chromosomes and chromosomal markers, no matter their size or mechanism of development, based only on their DNA content. Applying the same criteria, micronuclei that are classified microscopically into large/medium/small size[46], could be analyzed depending on their DNA content, and selectively sorted out. Thus, sorted micronuclei could be used for molecular analysis. It, thus, becomes obvious that the combination of analytical cytogenetic and molecular techniques can provide all the information required for the elucidation of the molecular events that are responsible

### ACKNOWLEDGMENTS

The author would like to thank Prof. B.D. Young for critically reading the present work and the staff of the Cancer Research U.K. Flow Cytometry facility for their valuable help. Furthermore, the present work was financially supported by a European Community Medical Research Division grant and by a British Council grant awarded to D. Matthopoulos to visit the Department of Medical Oncology, Imperial Cancer Research Fund, London.
